# Human vs Artificial Intelligence in Detecting Coronary Occlusions in ST-Elevation Myocardial Infarction: Retrospective Study

**DOI:** 10.2196/88399

**Published:** 2026-10-14

**Authors:** Aurelia Zimmerli, Marion Dupre, Quentin Liabot, C-Yasmine Sellah, David Meier, Adil Salihu, Victor Weerts, Vladimir Rubimbura, Olivier Roux, Olivier Muller, Olivier Hugli, Pierre-Nicolas Carron, Stephane Fournier

**Affiliations:** 1 Department of Cardiology, University Hospital of Lausanne, Rue du Bugnon 46, Lausanne, 1011, Switzerland, 41 795568205; 2 Hirslanden Klinik Im Park, Zurich, Switzerland; 3 Emergency Department, University Hospital of Lausanne, Lausanne, Switzerland

**Keywords:** artificial intelligence, AI, coronary artery disease, CAD, myocardial infarction, electrocardiogram, ECG

## Abstract

**Background:**

Occlusion myocardial infarction (OMI) on the electrocardiogram (ECG) can present as ST-elevation myocardial infarction (STEMI), non-STEMI, ST depression, or other patterns (eg, de Winter T waves and hyperacute T waves), posing a diagnostic challenge. Even experienced physicians may overlook OMI on ECG, leading to delays in catheterization laboratory activation. Recently, smartphone apps using AI have been shown to exhibit promising accuracy in ECG interpretation in large datasets.

**Objective:**

The objective of our study was to retrospectively compare the diagnostic performance of an AI-based application (PMCardio) with that of interventional cardiologists in interpreting nonpathognomonic ECGs that generated debates about diagnosis.

**Methods:**

In total, 33 ECGs that were diagnostically challenging upon patient admission to the emergency department were included. A group of 3 interventional cardiologists, blinded to the final diagnosis, independently assessed whether the ECG suggested OMI requiring immediate invasive coronary angiography. The diagnostic performance of the cardiologists was compared to that of the AI application, using the results of coronary angiography as the gold standard and considering any lesion with ≥90% stenosis as positive.

**Results:**

Of the 33 patients, 29 (88%) had a final diagnosis of cardiac origin for their chest pain, including 23 acute coronary syndromes, of which 13 (57%) were OMI. The 3 cardiologists achieved sensitivities of 62%, 85%, and 62%; negative predictive values (NPVs) of 74%, 83%, and 69%; specificities of 70%, 50%, and 55%; and positive predictive values (PPVs) of 57%, 52%, and 47%. Their overall misclassification rates were 33%, 36%, and 42%. In contrast, AI analysis demonstrated a sensitivity and NPV of 100%, a specificity of 60%, a PPV of 62%, and an overall misclassification rate of 24%.

**Conclusions:**

In this retrospective study of challenging ECGs, an AI application demonstrated better diagnostic performance of OMI as compared to cardiologists. These findings highlight the potential of AI to enhance diagnostic accuracy and support clinical decision-making.

## Introduction

In patients presenting with chest pain, electrocardiogram (ECG) allows one to distinguish between ST-elevation myocardial infarction (STEMI) and non-STEMI (NSTEMI). STEMI is generally caused by complete occlusion of a coronary artery, leading to ST-segment elevation on the ECG and requires immediate coronary angiography. Conversely, NSTEMI is typically associated with subtotal occlusion, resulting in ischemia-reperfusion phenomenon without ST-segment elevation. In this context, guidelines generally recommend coronary angiography to be performed within 24 hours [1].

However, this distinction has important limitations. Indeed, while STEMI criteria are highly specific (>95%), their sensitivity is much lower, at just above 40%, corresponding to a positive likelihood ratio of 12.5 but a negative likelihood ratio of only 0.58 [2]. On the other hand, more than 25% of NSTEMI patients are found to have a totally occluded culprit during coronary angiography [3]. Reliance on STEMI criteria alone to detect occlusion myocardial infarction (OMI) criteria is thus problematic, and a significant proportion of patients might receive delayed reperfusion, leading to larger infarcts and higher mortality rates.

OMI can present with different ECG patterns, including STEMI and NSTEMI, as well as atypical patterns sometimes referred to as “STEMI equivalents” [4,5]. Furthermore, new patterns are regularly described [6], adding to the diagnostic challenge.

Rapid and accurate diagnosis requires expertise in ECG interpretation in emergency settings, an expertise that is not always widely available depending on the timing (off-hours) and setting (primary care centers) of presentation. AI-based applications have been developed to aid ECG analysis, and recent studies have shown that AI can outperform both emergency physicians and interventional cardiologists in detecting coronary occlusion [7,8].

At Lausanne University Hospital (CHUV), ECGs of patients considered candidates for immediate reperfusion are transmitted from the prehospital setting or other hospital emergency departments (EDs) to a senior emergency physician, who decides whether urgent coronary angiography is warranted. When the ECG does not meet STEMI or STEMI-equivalent criteria, the on-call interventional cardiologist is consulted. This study aims to compare the diagnostic performance of an AI-based application (PMCardio) with that of interventional cardiologists in interpreting a consecutive series of challenging ECGs that do not meet STEMI criteria.

## Methods

### Data Collection

This is a single-center retrospective study conducted at CHUV. It represents the extension of a master’s thesis initially conducted as a quality improvement project. The original aim was to assess the use of a generic email system designed to dispatch ECGs of undetermined significance to the on-call interventional cardiologist. As this specific cohort was composed exclusively of ECGs for which a diagnostic uncertainty was present and whose final diagnosis had been adjudicated, it was used to evaluate the performance of an AI-based ECG interpretation tool.

All consecutive ECGs received by emergency physicians between February 26, 2019, and May 21, 2020, and submitted to the interventional cardiologist through this dispatch system were included. Only ECGs from patients who provided written informed consent and had a definitive established diagnosis were included in this analysis.

### Data Analysis

The ECGs were anonymized and independently evaluated by 3 experienced interventional cardiologists. Each cardiologist was asked to answer the following question: “If this ECG were associated with a compatible clinical presentation, would you activate the STEMI pathway for immediate invasive coronary angiography?” The same ECGs were uploaded as ECG images into the AI-based application PMCardio (PMcardio for Organizations, version 1.7.0; Powerful Medical).

PMCardio is a CE-marked AI medical device [9] that uses a deep neural network designed for the automated interpretation of 12-lead ECG waveforms. The initial version of the model (eOMI version 1) was trained on >18,000 ECGs from patients with chest pain and validated in an international study [7]. Subsequently, an updated version of the model (aOMI version 1) was developed and recalibrated on >200,000 ECGs to improve performance in identifying STEMI and STEMI-equivalent patterns associated with active coronary occlusion requiring emergent reperfusion. Across development iterations, the model was trained on a large multinational dataset comprising >200,000 ECG recordings linked to clinical outcomes and angiographic findings. The reference standard used for model training consisted of angiographically confirmed occlusive myocardial infarction, defined as the identification of a culprit vessel with either total occlusion or critical stenosis associated with acute myocardial infarction and biomarker elevation, as adjudicated using the full clinical record. In the present validation study, ECGs were analyzed using the PMcardio for Organizations smartphone app (version 1.7.0). ECG tracings were uploaded as photographs of printed ECGs captured via smartphone, and the app automatically digitized the ECG images into waveform signals using a validated AI-based ECG digitization pipeline before applying the diagnostic model [10]. Importantly, the datasets used for model development and training came from a separate study and were entirely independent of the validation cohort used in the present study. No overlap or data leakage occurred between the development or training datasets and the validation cohort. The present analysis therefore represents an external evaluation of the algorithm in a separate cohort of diagnostically challenging ECGs, minimizing the risk of overfitting.

The diagnostic concordance of cardiologists and the AI was assessed against the final clinical diagnosis of OMI, with coronary angiography serving as the gold standard and lesions with ≥90% stenosis classified as positive. The angiographic report included assessments of culprit vessel identification and thrombolysis in myocardial infarction (TIMI) flow. Final diagnostic adjudication was corroborated using the discharge summary, which incorporated troponin values and other clinical and imaging data.

### Statistical Analysis

All statistical analyses were conducted using SPSS (version 24.0; IBM Corp). Figures were generated using Prism (version 7.0a; GraphPad Software). General patient data are presented as mean (SD) or median (IQR), depending on the results of normality tests. Qualitative analyses were performed to categorize ECG diagnostic uncertainties into distinct groups. The misclassification rate was defined as the proportion of incorrectly classified cases, calculated as the sum of false-positive and false-negative results divided by the total number of cases. Conversely, diagnostic accuracy was defined as the proportion of correctly classified cases, calculated as the sum of true-positive and true-negative results divided by the total number of cases. To evaluate the differences in diagnostic performance between the AI application and the cardiologists, we used the McNemar test based on the consensus adjudication of the 3 cardiologists rather than individual reader assessments, as this approach reduces interreader variability and better reflects real-world expert evaluation of ambiguous ECGs.

### Ethical Considerations

This study received ethics approval from the Cantonal Ethics Committee of Vaud, Switzerland (project ID: 2020-01451). Written informed consent was obtained from all included patients. The consent covered secondary analysis of clinical data. All ECGs were anonymized prior to analysis. No directly identifiable data were accessible to investigators. Participants did not receive any financial compensation for their participation in the study.

## Results

### Overview

A total of 62 ECGs were initially collected between February 26, 2019, and May 21, 2020. Among them, 25 patients had previously provided general consent for the use of their clinical data for research purposes. For the remaining patients, additional consent was sought by mail when contact information was available. When necessary, referring hospitals were contacted to retrieve missing contact details. Overall, 30 consent forms were sent, resulting in 9 additional patients providing consent, 4 refusals, and 17 nonresponses. Thus, a total of 34 patients consented to inclusion. Final clinical diagnoses were obtained from electronic medical records or by contacting referring institutions when necessary. One patient could not be reached, resulting in 33 patients with complete data included in the final analysis (Figure 1). The study included 21 men (63%) and 12 women (36%), with a mean age of 69 (SD 8.0) years for men and 74 (SD 3.25) years for women.

**Figure 1.**
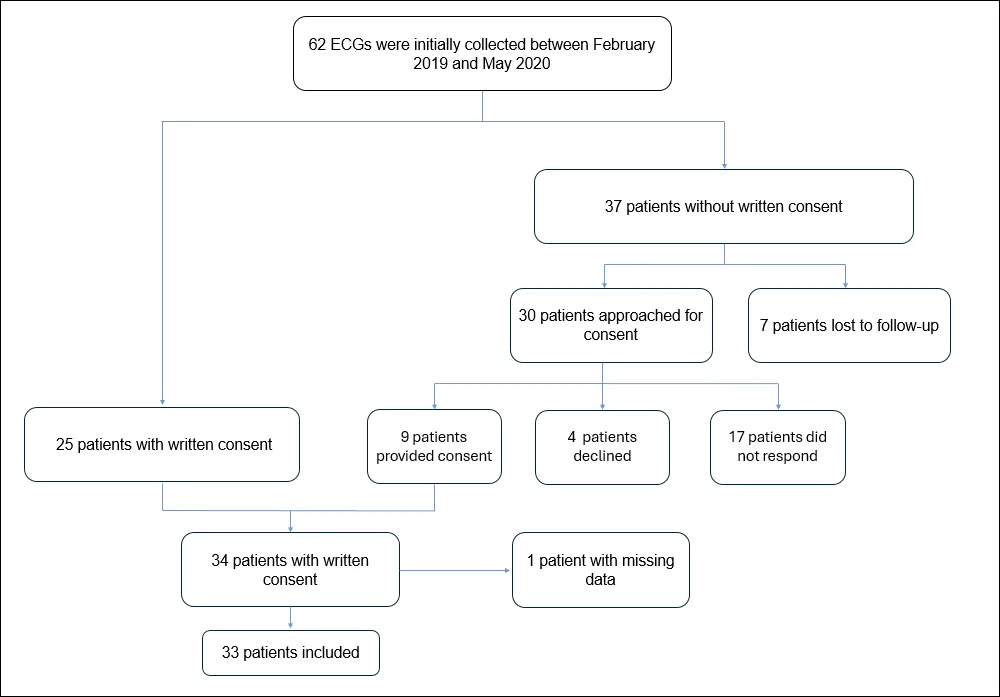
Flow diagram of patient selection. A total of 62 electrocardiograms (ECGs) were collected between February 2019 and May 2020. After additional consent procedures, 34 patients provided consent. Final diagnoses were obtained from medical records or referring institutions. One patient was excluded due to missing data, resulting in 33 patients included in the final analysis.

### Final Diagnosis

Among 33 patients, 29 (88%) were confirmed to have a cardiac origin of their symptoms, including 23 acute coronary syndromes (ACS), of which 13 (57%) were OMI. The remaining 4 patients were diagnosed with extracardiac conditions (Figure 2).

**Figure 2.**
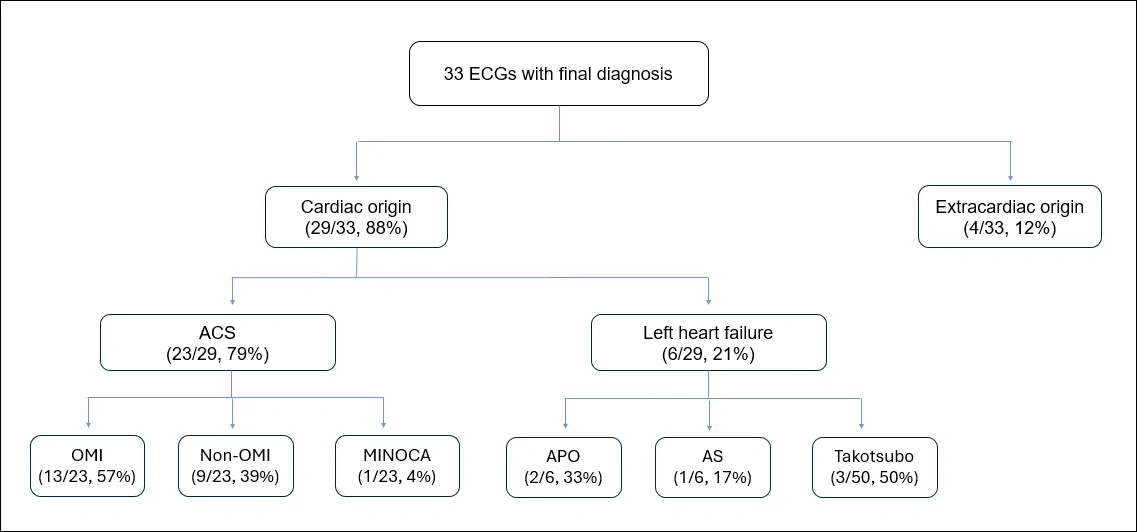
Flow diagram of the final diagnosis in a retrospective study of 33 patients undergoing electrocardiogram (ECG) evaluation. The study included patients with suspected cardiac events at Lausanne University Hospital between February 2019 and May 2020. ACS: acute coronary syndrome; APO: acute pulmonary oedema; AS: aortic stenosis; MINOCA: myocardial infarction with nonobstructive coronary arteries; OMI: occlusion myocardial infarction.

### Analysis by Interventional Cardiologists

Cardiologist 1 correctly identified 8 out of 13 patients with an occluded or nearly occluded vessel (≥90% stenosis), corresponding to a sensitivity of 62%. He recommended an emergency procedure in 14 patients, among whom 8 had an occlusion or near-occlusion, yielding a positive predictive value (PPV) of 57%. Among the 20 patients without such lesions, he refrained from indicating an urgent catheterization in 14 cases, resulting in a specificity of 70% (95% CI 50%‐90%). Finally, in the 19 cases where no emergency angiography was advised, 5 patients were later found to have an occluded or nearly occluded vessel, leading to a negative predictive value (NPV) of 74% (95% CI 54%‐94%; Figure 3).

**Figure 3.**
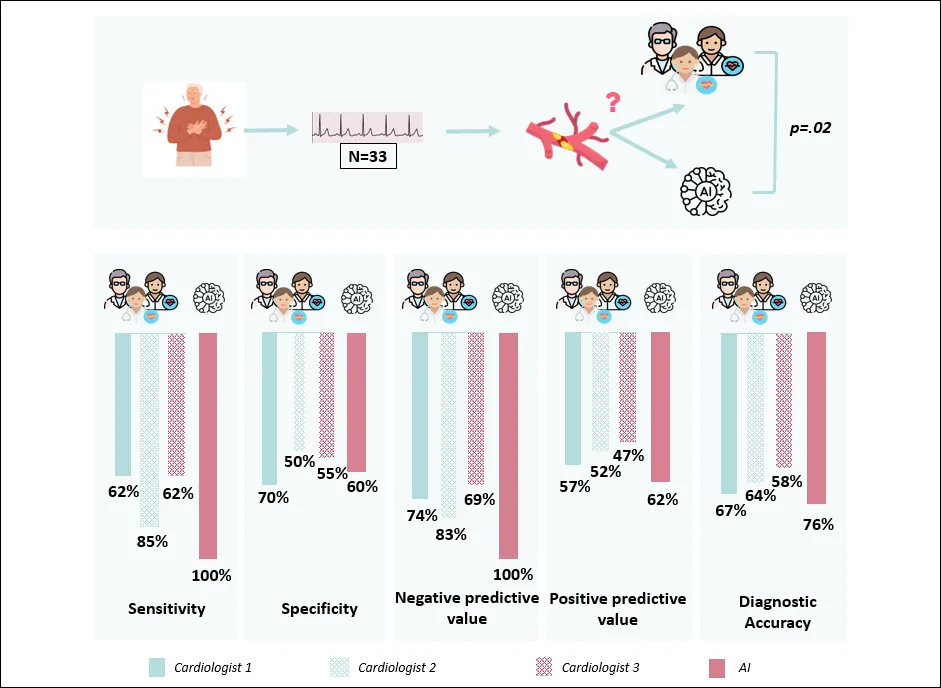
The diagnostic performance of cardiologists vs an AI algorithm for detecting occlusion myocardial infarction (OMI) in a retrospective study of 33 difficult-to-interpret electrocardiograms (ECGs). Using the exact binomial version of the McNemar test, the AI system performed significantly better than the consensus diagnosis of cardiologists (*P*=.02).

Cardiologist 2 correctly identified 11 out of 13 patients with an occluded or nearly occluded vessel (≥90% stenosis), corresponding to a sensitivity of 85% (95% CI 64%-100%). He recommended an emergency procedure in 21 patients, among whom 11 had an occlusion or near-occlusion, yielding a PPV of 52% (95% CI 30%-74%). Among the 20 patients without such lesions, he refrained from indicating an urgent catheterization in 10 cases, resulting in a specificity of 50% (95% CI 34%-76%). Finally, in the 12 cases where no emergency angiography was advised, 2 patients were later found to have an occluded or nearly occluded vessel, leading to an NPV of 83% (95% CI 62%-100%; Figure 3).

Cardiologist 3 correctly identified 8 out of 13 patients with an occluded or nearly occluded vessel (≥90% stenosis), corresponding to a sensitivity of 62% (95% CI 35%-88%). He recommended an emergency procedure in 17 patients, among whom 8 had an occlusion or near-occlusion, yielding a PPV of 47% (95% CI 23%-71%). Among the 20 patients without such lesions, he refrained from indicating an urgent catheterization in 11 cases, resulting in a specificity of 55% (95% CI 34%-76%). Finally, in the 16 cases where no emergency angiography was advised, 5 patients were later found to have an occluded or nearly occluded vessel, leading to an NPV of 69% (95% CI 46%-92%; Figure 3).

When comparing the diagnosis provided by the 3 cardiologists, all 3 agreed on the same diagnosis for 18 patients, while partial agreement (2 of 3 cardiologists) was observed for 15 patients.

### Analyses by the AI-Based Application

The AI-based application identified 13 out of 13 patients with an OMI, corresponding to a sensitivity of 100% (95% CI 75%-100%). An emergency procedure was recommended in 21 patients, yielding a PPV of 62% (95% CI 39%-85%). Among the 20 patients without OMI, urgent catheterization was recommended in 8 cases, resulting in a specificity of 60% (95% CI 38%-82%) and an NPV of 100% (95% CI 73%-100%; Figure 3).

Furthermore, AI demonstrated a misclassification rate of 24%, whereas the interventional cardiologists had misclassification rates ranging from 33% to 42%.

To compare human performance, a consensus diagnosis among cardiologists was adjudicated on majority vote in cases of disagreement. This analysis showed that the AI system performed significantly better than the cardiologists (*P*=.02).

### Misclassified Pathological ECG

Among the ECGs analyzed, 1 case was particularly illustrative of the diagnostic divergence between OMI and its ECG manifestations (Figure 4). Coronary angiography subsequently revealed an occlusion of a marginal branch artery. The AI-based application correctly identified the ECG as an OMI and recommended immediate coronary angiography. Conversely, none of the interventional cardiologists interpreted the ECG as warranting urgent invasive evaluation.

**Figure 4.**
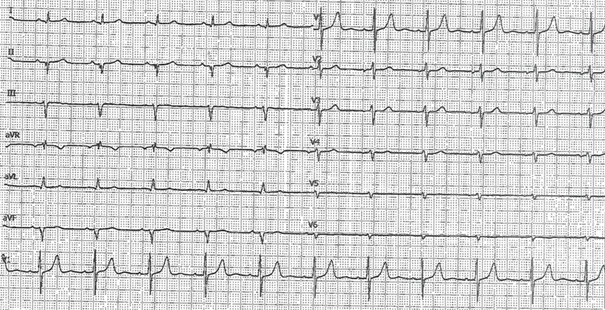
Representative electrocardiogram (ECG) from a patient included in the study cohort with angiographically confirmed occlusion of a marginal branch artery. In this case, none of the 3 cardiologists identified the diagnosis on ECG, whereas the AI algorithm correctly detected occlusion myocardial infarction.

## Discussion

### Principal Findings

In this retrospective study of 33 real-life challenging ECGs, 13 (39%) were confirmed as OMI by coronary angiography. The sensitivity of the cardiologist for detecting OMI ranged from 62% to 85%, while the AI-based application achieved a sensitivity of 100%. The AI-based application also had a lower misclassification rate (24%) compared to cardiologists (33%-42%), demonstrating a superior diagnostic performance. Notably, the AI application did not miss any OMI cases, a finding of particular clinical relevance given the consequences of delayed reperfusion. This further supports the importance of classifying patients presenting with chest pain as OMI or non-OMI, rather than relying solely on conventional ECG criteria, to guide the timing of coronary revascularization [11].

### Clinical Implications

The higher number of false positives observed among cardiologists compared to the AI-based tool should be interpreted with caution. The misclassification rate was used as a global measure of diagnostic error; however, this metric combines false-positive and false-negative results and therefore assumes equal clinical weight between them. In the context of suspected OMI, these errors have different implications. False negatives may lead to delayed reperfusion and potentially worse outcomes, whereas false positives mainly result in unnecessary catheterization laboratory activation and increased resource use. For this reason, sensitivity and NPV remain particularly important metrics when evaluating diagnostic tools intended to support early detection of OMI. In our analysis, any case in which an OMI was suspected where angiogram showed a stenosis <90% was classified as a false positive. However, this binary categorization does not necessarily capture the full clinical rationale underlying the decision to proceed with an invasive strategy. Moreover, some occlusions can spontaneously recanalize, notably by responding to antiplatelet and anticoagulant therapy (eg, aspirin and heparin) administered before coronary angiography. This dynamic can lead to underestimation of the initial severity of the coronary lesion. Taken together, these considerations suggest that the reported false-positive rate, especially among cardiologists, should be interpreted with clinical nuance and not as a strict marker of diagnostic error.

### Comparison With Prior Work

This study was conducted in a highly selected cohort of ECGs that prompted real-time consultation with an interventional cardiologist. As such, the prevalence of OMI in our sample (13/33, 39%) is substantially higher than that observed in an unselected ED population. Therefore, our results should be interpreted within the specific context of diagnostically ambiguous ECGs rather than as a reflection of screening performance in all-comers chest pain populations.

AI might play a central role in the analysis of ECGs in patients with suspected ACS [7]. Diagnosis of OMI on the ECG remains a complex challenge, as studies have shown that approximately one-third of OMI cases do not present with a typical ST-segment elevation on the initial ECG [12]. Moreover, only 49% of OMI cases are recognized by cardiologists, leading to undertreatment of a time-sensitive and potentially lethal pathology [13,14]. Our study showed that in this specific population with complex ECGs, AI demonstrated higher sensitivity than cardiologists. Furthermore, cardiologists exhibited significant interindividual variability, with 18 ECGs receiving the same diagnosis from all 3 specialists. These findings underscore the potential of AI as a valuable tool in ECG interpretation, particularly in detecting subtle or ambiguous signs of OMI. By improving diagnostic accuracy and assisting clinical decision-making, AI has the potential to enhance patient outcomes, making its integration into clinical practice highly relevant. Our study contributes to the growing body of literature supporting the use of AI for OMI detection and provides evidence of its validity in a local clinical setting.

### Limitations

This study has several limitations. First, the small sample size is due to the specific population defined by diagnostically challenging ECGs, adjudicated final diagnosis, and written informed consent. As a result, critically ill patients were excluded, which may have introduced survivorship bias. These findings should therefore be validated in a larger and more representative cohort. Second, no clinical data were available for analysis, including patient presentation (symptom onset, duration, and characteristics), which may have impacted the diagnostic performance of the cardiologists and does not fully reflect real-world clinical conditions. Additionally, the quality of some ECGs was suboptimal, with incomplete tracings. Finally, OMI defined by ≥90% stenosis provides an objective angiographic benchmark but may oversimplify clinical reality. Unstable thrombotic lesions with <90% stenosis or reduced TIMI flow can justify urgent reperfusion. Therefore, some false positives may represent appropriate clinical decisions rather than diagnostic errors.

### Conclusions

AI-based ECG interpretation showed a higher sensitivity and a lower misclassification rate compared to interventional cardiologists for the detection of OMI in this cohort of challenging ECGs. These findings suggest that AI may represent a valuable adjunctive tool to support clinical decision-making. Further studies in larger and more representative populations are needed to confirm these results and better define the role of AI in emergency cardiac care.

## Abbreviations

ACS: acute coronary syndrome
CHUV: Lausanne University Hospital
ECG: electrocardiogram
ED: emergency department
NPV: negative predictive value
NSTEMI: non–ST-elevation myocardial infarction
OMI: occlusion myocardial infarction
PPV: positive predictive value
STEMI: ST-elevation myocardial infarction
TIMI: thrombolysis in myocardial infarction
